# Molecular Understanding of HIV-1 Latency

**DOI:** 10.1155/2012/574967

**Published:** 2012-04-04

**Authors:** W. Abbas, G. Herbein

**Affiliations:** Department of Virology, University of Franche-Comte, 2 Place Saint-Jacques, EA 4266, IFR 133, CHU Besancon, 25030 Besançon Cedex, France

## Abstract

The introduction of highly active antiretroviral therapy (HAART) has been an important breakthrough in the treatment of HIV-1 infection and has also a powerful tool to upset the equilibrium of viral production and HIV-1 pathogenesis. Despite the advent of potent combinations of this therapy, the long-lived HIV-1 reservoirs like cells from monocyte-macrophage lineage and resting memory CD4+ T cells which are established early during primary infection constitute a major obstacle to virus eradication. Further HAART interruption leads to immediate rebound viremia from latent reservoirs. This paper focuses on the essentials of the molecular mechanisms for the establishment of HIV-1 latency with special concern to present and future possible treatment strategies to completely purge and target viral persistence in the reservoirs.

## 1. Introduction

 Infection with HIV-1, which was first isolated in 1983, causes AIDS, a syndrome that was first reported in 1981 [1]. The HIV-1 pandemic represents one of the great plagues in the history of mankind and a major challenge for medicine, public health, and medical research [2]. The majority of people living with HIV-1 belong to low- and middle-income countries. For example, sub-Saharan Africa accounts for two third of all infected people with HIV-1, where in few countries more than one in five adults are infected with HIV. South and south East Asia have second highest number of people living with HIV-1. Furthermore the epidemic is spreading most rapidly in Eastern Europe and central Asia, where the number of people living with HIV increased by 54.2% between 2001 and 2009. UNAIDS estimated that 33.3 million people were infected with HIV at the end 2009 compared to 26.2 million in 1999, a 27% increase in HIV infection. Each year 2.6 million people are infected with HIV-1 and 1.8 million die of AIDS (UNAIDS 2010). Much has been learned about the science of AIDS and continuous research has allowed the development of 25 different active compounds belonging to six different drug families shifting the HIV-1 infection from a fatal illness into a chronic disease [3, 4].

**Figure 1:**
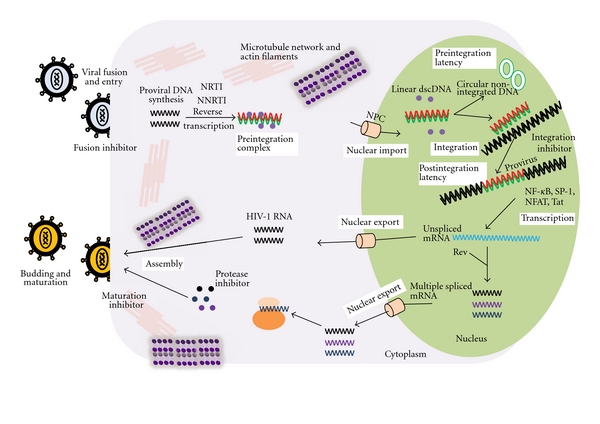
Schematic representation of HIV-1 life cycle and latency with current and possible targets for antiviral intervention. The HIV-1 life cycle begins with the attachment of virus to the receptor (CD4) and coreceptor (CXCR4 or CCR5), followed by fusion with target cell membrane. After virus entry, the viral nucleocapsid enter the cytoplasm, undergoes reverse transcription and then uses cytoplasmic dynein to move towards the nuclear pore complex. The preintegration complex is transported into nucleus through NPC, and then dscDNA either circulizes as one or two LTR containing circle or is integrated into a host cell chromosome. After integration the provirus remains quiescent in post integration latent state. On activation the viral genome is transcribed by cellular transcription factors, spliced mRNA are transported into cytoplasm where viral mRNA translated into regulatory and structural viral proteins. New virions assemble and bud through cell membrane, maturing through the activity of viral protease. The different classes of antiretroviral drugs are available. Fusion or HIV co-receptor inhibitors inhibit the entry of virions into a new target cell. The step of reverse transcriptase can be targeted, using nucleoside analogues or non-nucleoside reverse transcriptase inhibitors (NRTI and NNRTI, resp.). The HIV-1 integrase inhibitors inhibit the strand transfer reaction in the integration process, a crucial step in the stable maintenance of the viral genome, as well as efficient viral gene expression and replication. The class of protease inhibitors interferes with the last stage of viral life cycle which results in the production of noninfectious viral particles. The HIV maturation inhibitors disrupt a late step in HIV-1 Gag processing.

 HIV-1 life cycle can be categorized into two phases. The early stage occurs between entry into host cells and integration into its genome (Figure 1). The late phase occurs from the state of integrated provirus to full viral replication [5]. Similarly two types of viral latency can be differentiated: preintegration latency results in generation of different forms of viral DNA before integration, whereas postintegration latency refers to the lack of viral replication after the insertion of viral DNA into host genome [6]. Virus enters through successive interactions with CD4 and CXC chemokine receptor type 4 (CXCR4) or CC chemokine receptor type 5 (CCR5); as a consequence HIV-1 core (diploid single strand positive sense RNA, tRNA primers, viral protease, retrotranscriptase, and integrase) is released into cytoplasm [7, 8]. After reverse transcription, the preintegration complex (linear dsDNA, integrase, matrix protein, retrotranscriptase, viral protein r and various host proteins) transportation into nucleus is mediated by microtubule and dynein, thereby allowing the infection of resting, nondividing cells. Linear dsDNA either integrates into host cell chromosomes or circulartes as one or two long terminal repeat (LTR) containing circles [9, 10].

Activation of host cells induces the binding of transcriptional preinitiation complex to enhancer elements in the 5′LTR proximal promoter that gathers essential host transcription factors, such as NF-*κ*B, NFAT, AP-1 and SP1 which transmit activation signals to basal transcription machinery and promote the binding of RNA polymerase II to the TATA box to initiate transcription [11–13]. Transactivation response element (TAR), a 59-nucleotide stem loop structure, is then formed at 5′ end of nascent viral transcript that creates the binding site for viral transactivator (Tat) which promotes efficient elongation of viral transcripts by recruiting the positive transcription elongation factor b (PTEFb), thereby enhancing the functional capacity of RNAPII [14]. Viral regulatory protein (Rev) regulates the processing, nuclear cytoplasmic transport, and splicing of HIV mRNA. HIV-1 large precursor proteins assemble to create viable particles budding off the cell and are processed into mature proteins [15].

 In 1996, the introduction of highly active antiretroviral therapy (a combination of three or more potent anti-HIV-1 drugs targeting different steps of viral life cycle) has greatly extended the survival and stabilized the AIDS pandemic on global scale (Table 1). This therapy can reduce the plasma virus levels below detection limits (<20 copies/mL) [16, 17]. However a residual viremia is still detected in patients on HAART with very sensitive methods. Furthermore, HIV-1 reverts to measurable plasma level in less than two weeks when HAART is interrupted [18]. These observations suggest that HAART cannot totally eliminate HIV. The virus persistence in cellular reservoirs because of viral latency, cryptic ongoing replication, or poor drug penetration represents the major obstacles for its eradication [19].

HIV-1 replicates preferentially in activated CD4^+^ T cells but these cells generally survive only for few days after infection. Hardly, an infected CD4+ lymphoblast survives long enough to revert back to a resting memory state [20–22]. Furthermore, HIV-1 gene expression is largely suppressed as cells undergo this transition because HIV-1 LTR transcription is heavily dependent on the inducible host transcription factors (NF-*κ*B, NFAT, and AP-1, etc.) that are excluded from the nuclei of resting cells [23, 24]. The result is a stably integrated but transcriptionally silent provirus in a memory T cell, whose function is to survive for longer period of time. If the cell is activated by cytokine or other stimuli, it can begin to produce virus; otherwise the virus persists as integrated DNA, unaffected by antiretroviral drug [25]. During rebound viremia (when HAART was interrupted) the virus could be detected from another reservoir (other than resting CD4+ T cells) from peripheral blood monocytes, dendritic cells, and macrophages in lymph node, can be infected latently, and therefore contribute to the viral persistence [26–28]. Furthermore several features make cells from macrophages lineage potential HIV reservoir. The viral particles produced in macrophages are budding into intracytoplasmic compartments which may represent one of the favored sites for HIV replication [29]. Furthermore cells from macrophages lineage are also more resistant to apoptosis and cytopathic effects, harbor virus for longer period of time, and produce virus throughout their life span even in the microglial brain cells [30].

HAART results in a four-phase decline of viremia [31]: first an initial rapid loss of virus due to clearance of infected activated CD4+ T cells surviving about one day because of viral cytopathic effects or host cytolytic effector mechanism; the second slower phase of viral decay in infected macrophages, partially activated CD4+ T cells, and follicular dendritic cells (FDCs) due to the clearance of several cell population with a half-life of one to four weeks; the third phase of decay corresponding to cells with half-life of several weeks (memory cells); and the fourth phase with no appreciable decline, caused by the activation of resting memory CD4+ T cells [32]. During the fourth phase, HIV-1 plasma level normally ranges from 1 to 5 copies (RNA per mL) and can be detected by extremely sensitive RT-PCR assays [33].

A 60-year uninterrupted HAART has been estimated being necessary to eradicate the latent reservoirs. The lifelong HAART treatment is today a necessary evil because of its association with many metabolic disorders and toxicities [34, 35]. Moreover, its interruption leads to rapid viral rebound, attributable to the persistence latently infected memory CD4+ T cells. Cells from latently infected reservoirs (cell where virus persists with more stable kinetics than main pool of actively replicating virus) are immunologically alike from uninfected cells and are insensitive to immune clearance and HAART [36]. To address the persistence of transcriptionally silent but replication competent HIV reservoirs, the first approach could be to strengthen and intensify HAART by introducing new viral inhibitors. Secondly, if therapeutic goal is virus eradication, then novel strategies need to be adopted to target and clear the latent reservoirs by inducing HIV-1 replication in latently infected cells, while maintaining or intensifying HAART in order to prevent spreading of new infections [37].

## 2. Molecular Insights into HIV-1 Latency

 Two forms of viral latency have been seen on the basis whether or not HIV-1 genome has integrated into the host cell genomes: preintegration and postintegration latency [38]. Preintegration latency results in partial or complete blockade of viral life cycle prior to integration of virus genome into host genome. It could result from incomplete reverse transcription or from restriction by factors such as APOBEC3G (cellular deoxycytidinedeaminase whose function can be counteracted by viral vif protein) [39, 40]. Further the preintegration latency does not appear to be of clinical significance because unintegrated forms persist in the cytoplasm for only one day and cannot account for long-term latently infected CD4+ T-cell reservoirs but this unintegrated form of DNA remains stable for at least one month in nondividing metabolically active macrophages [41, 42]. Postintegration latency occurs when HIV-1 genome integrated into host genome is reversibly silenced and is limited only by the life span of the infected cells and its progeny. Most mechanisms to maintain HIV-1 latency operate at transcriptional level.

### 2.1. The Site and Orientation of Integration

 HIV-1 latency mostly operates at the transcriptional level; for example, the chromosome environment at the site of integration and availability of viral and host factors can have influence on viral latency [10]. HIV-1 integrates into the host chromosomal DNA in nonrandom manner. Specific sequences at the ends of dscDNA are required to target PIC predominantly to the intronic regions of the actively transcribed genes [43]. One study of the integration sites in purified resting CD4+ T cells from the patients on HAART found majority of provirus (93%) located within the coding regions of host gene, probably owing to the increased chromatin accessibility of these regions [44]. The finding that latent HIV-1 proviruses integrate in actively transcribed regions may seem paradoxical considering the establishment of transcriptional latent state [45]. However, the viral replication from these proviruses can suffer from intense transcriptional interference because of the orientation of the proviruses or their proximity to a stronger host gene promoter [46].

 The steric hindrance occurs: when the provirus integrates in the same transcriptional orientation as the host gene, read-through transcription from upstream promoter displaces key transcription factors from HIV-1 promoter and prevents the assembly of the preinitiation complex on the viral promoter, thereby hindering HIV-1 transcription [47, 48]. These transcriptional interferences could be reversed by inhibiting the upstream transcription or by cooperatively activating viral transcription initiation and elongation [49]. Furthermore, integrated provirus suffering from transcriptional interference becomes transcriptionally active following Tat expression, and this provirus can switch off the transcription of the host genes within which it has integrated or can allow the coexistence of expression of both host and viral genes [50].

 Promoter occlusion occurs when provirus integrated in opposite orientation to the host gene may lead to the collision of two RNAPII complexes during elongation, which can lead the premature termination of the transcription of one or both complexes [45, 51]. Convergent transcription may also allow for the elongation of both viral DNA strands which results in the formation of double-stranded RNAs, might lead to RNA-interference, RNA directed DNA methylation or generation of antisense RNA [52, 53]. Furthermore, the phenomenon of enhancer trapping can occur when enhancer of one gene is placed out of context near the promoter of the second gene. Taken together, the orientation-dependent regulation is highly variable and relies on the 5′LTR occupancy and on the rate of host gene elongation [54, 55].

### 2.2. Availability of Host Cell Transcription Factors and HIV-1 Viral Proteins

HIV-1 gene expression is strongly dependent on host cell transcription machinery and the lack of host transcriptional activator or the presence of host transcription repressors also influences the viral latency. The 5′LTR functions as an HIV-1 promoter and contains several DNA binding sites for various cellular transcription factors such as SP1 and NF-*κ*B which are required for viral replication, whereas other sites, such as those binding NFAT, LEF-1, COUP-TF, ets-1, USF, and AP-1, enhance transcription without being indispensable [56–58]. The p50/p65 NF-*κ*B heterodimer is sequestered into the cytoplasm in unstimulated cells through its interaction with an inhibitory protein of the family of NF-*κ*B inhibitors (I*κ*Bs) [59]. Following cellular activation, phosphorylation of I*κ*B by I*κ*B kinase (IKK) results in its dissociation from NF-*κ*B, NF-*κ*B translocation into the nucleus, and transcription of NF-*κ*B-dependent genes [60]. On the contrary, the NF-*κ*B p50/p50 homodimers, which lack the transactivation domain, recruit the histone deacetylase HDAC-1 to the LTR, leading to local histone deacetylation and to a repressive chromatin environment on the HIV-1 5′LTR in HIV-infected cells [61, 62]. Following T-cell activation, p50/p50 homodimers are uprooted by the p50/p65 heterodimers, which recruit histone acetyltransferases (CBP and p300) to enhance the viral replication [24, 63]. Furthermore, the p65 subunit of NF-*κ*B stimulates transcriptional elongation by interacting with RNAPII complexes of cdk7/TFIIH and pTEFb which in turn phosphorylate the serine-5 and serine-2 residues, respectively, in the carboxyl terminal domain (CTD) of RNAPII for the efficient transcription elongation [64, 65].

As far as NFAT is concerned, T-cell activation dephosphorylates cytoplasmic NFAT via PKC pathway and translocates into the nucleus where it interacts with 5′LTR at the sites overlapping the U3 NF-*κ*B binding site and thus promotes the chromatin remodeling by recruiting transcriptional coactivator like CBP and p300 [66, 67]. Further the AP-1 complex, composed of Jun, Fos, and ATF family members, having three binding sites in HIV-1 5′LTR, cooperates with NFAT to activate HIV-1 transcription through U3 NF-*κ*B/NFAT binding sites [68, 69]. In addition to host cell transcription factors, HIV-1 transcription is boosted by viral protein like Tat [70]. Tat interacts with the cis-acting RNA element TAR (transacitivation response element) present at the 5′ of viral transcripts. The inhibition of Tat also induces latency because in its absence, transcription is initiated but blocked at the promoter in the early stage of elongation due to the repressive chromatin environment [71, 72]. Tat activity is regulated mainly through the acetylation of Lys28 and Lys50 [73]. Tat acetylation by PCAF on Lys28 enhances the recruitment of pTEF at 5′ end of nascent viral transcripts promoting efficient elongation, whereas acetylation of Lys50 by CBP promotes the dissociation of Tat from Tat-cyclin T complex, allowing its interaction with PCAF and Tat-PCAF complex recruiting to the elongating RNAPII [74–76]. Some cellular protein affects the acetylation state of Tat modulating its activity. Sirtuin 1, a class III HDAC, acts as specific Tat deacetylase, thus increasing the quantity of Tat that is available to act as a transcriptional activator [77]. Further CDK9, a component of pTEFb, is acetylated by hGCN5 and PCAF, reducing the transcriptional activity of pTEFb and promoting HIV-1 latency [78].

In addition to transcription factors and their regulators, specific restriction factors exist to defend host cell against retroviral infection. For example, APOBEC3G impairs early phases of HIV-1 life cycle and may induce latency. APOBEC3G strongly inhibits HIV-1 replication in CD4+ T cells by inducing C to U conversions in the viral strand DNA during reverse transcription [79]. This viral replication inhibitory effect of APOBEC3G is only present in resting cells, where it exists as an active, low molecular mass ribonuleoprotein complex [80]. T-cell activation induces the shift from an active low molecular mass to inactive high molecular mass form of APOBEC3G that cannot restrict viral infection. This inactive form of APOBEC3G can be found in tissue resident naïve or memory CD4+ T cells, which are permissive to HIV-1 infection [81].

### 2.3. The Chromatin Organization and Epigenetic Regulation of HIV-1 Promoter

HIV-1 promoter activity depends on the chromatin environment where two nucleosomes, namely, nuc-0 and nuc-1, are precisely positioned at the viral promoter in latently infected cell lines and impose a block to transcriptional elongation. Nuc-1 nucleosome, located immediately downstream the transcription initiation site, impedes the LTR activity [82, 83]. Epigenetic modification and disruption of nucleosome, nuc-1, are required for LTR-driven transcription activation and viral gene expression [84]. The chromatin organization can be modulated through a variety of mechanisms, including posttranslational covalent modifications of histone tails and ATP-dependent, chromatin remodeling events [85, 86]. Histone modification (i.e., acetylation, methylation, phosphorylation, sumoylation, ADP-ribosylation and ubiquitination) can influence the gene expressions, which are all reversible and localized to N- and C-terminus of histone tails [87, 88]. Hypoacetylation of histones by histone deacetylase (HDACs) correlates with transcription repression, whereas hyperacetylation by histone acetyltransferase (HATs) induces the transcription activation [89].

The silent proviral 5′LTR can be activated from postintegration latency by cell treatment with a variety of stimuli, including cytokines like TNF-*α* and IL-6, antibodies (anti-CD3 and -CD28 stimulation) phorbol esters (PMA, PHA, prostratin), or by viral proteins (Tat and Nef). The nucleosome nuc-1, located immediately downstream of transcription start site, is specifically remodeled following IL-6, TNF, or PMA treatment, and this event is specifically correlated with the activation of HIV-1 gene expression [82, 84]. Furthermore, HIV-1 transcriptional activation was shown to occur following treatment with HDAC inhibitors (HDACIs) such as trichostatin A (TSA), trapoxin (TPX), valproic acid (VPA), and sodium butyrate (Na But), suggesting that during latency nuc-1 is constitutively deacetylated by HDACs [90, 91]. The HDACI-mediated transcriptional activation is accompanied by specific remodeling of nuc-1 and by an increased acetylation of H3K4 and H4K4 in the promoter region [92].

Several transcription factors such as ying and yang (YY1) and late SV40 factor (LSF; also known as TFCP2) repress the HIV-1 replication by recruiting HDAC1 to repressor complex sequence located at position −10 to +27 nucleotides in the LTR [93, 94]. Other host transcription factors, such as AP-4 (activating protein-4), NF-*κ*B p50/p50 homodimers, and CBF-1 (C-promoter binding factor-1), can also recruit HDACs to the LTR and inhibit viral transcription [61, 95]. By contrast viral proteins like Tat and several cytokines and HDAC inhibitors decrease HDACs occupancy at the repressor complex sequence and activate the transcription at 5′LTR by recruiting factors with HAT activity such as CREB binding protein (CBP), CBP-associated factors (PCAFs), and human general control of amino acids synthesis protein 5, which induces nucleosome hyperacetylation in cell lines [49, 96]. Similarly, in the absence of Tat, LTR-associated nucleosomes are hypoacetylated, and viral gene expression is silenced, contributing to viral latency. HDAC inhibitors are not sole factors to induce transcription; host factors such as NF-*κ*B, NF-AT, and SP-1 must also be recruited to the 5′LTR [97, 98].

Generally, the histone acetylation is associated with gene activation while histone methylation can be associated with both activation and silencing. For example, methylation of histone 3 at lysine 4 and histone 3 at lysine 36 is found in active genes whereas methylation of histone 3 at lysine 9 and 27 and histone 4 at lysine 20 is associated with gene silencing [99]. The histone 3 at lysine 9 methylation that is mediated by SUV39H1 (suppressor of variegation 3–9 homologue 1) has been correlated with heterochromatin assembly by recruiting HP1*ϒ* (heterochromatin protein 1 homologue-gama), resulting in HIV-1 silencing [96]. The transcription factor COUP-TF interacting protein 2 (CTIP2) plays an essential role in promoting viral latency in microglial cells by recruiting a chromatin modifying enzyme complex and by establishing a heterochromatic environment at the HIV-1 promoter [100]. Actually, the CTIP2 recruits HDAC1, HDAC2, SUV39H1, and HP1 proteins to establish a heterochromatic environment that leads to HIV-1 silencing in several cell lines [101]. Finally, by altering histones, recruiting other chromatin remodeling factors, and modifying the activity of certain transcription factors, HDACs appear to be critical for epigenetic repression of HIV-1 transcription and for the maintenance of viral latency [102].

### 2.4. Posttranscriptional Latency and MicroRNAs

MiRNAs are single-stranded noncoding RNAs of 19 to 25 nucleotides in length that function as posttranscriptional regulator and introduce a new level of complexity to virus-host interplay [103, 104]. Further miRNAs can also regulate the gene expression at the epigenetic level by remodeling chromatin surrounding [105]. Several cellular miRNAs (miR-28, miR-125b, miR-150, miR-223, and miR-382) control HIV-1 replication by targeting all spliced or unspliced HIV-1 mRNA except Nef coding mRNA [106]. These cellular miRNAs are enriched in resting CD4+ T cells and inhibit the translation of almost all HIV-1-encoded proteins contributing to viral latency [107]. Furthermore, viral genome produces viral interferences RNAs that can target the viral RNAs, cellular mRNAs, and host miRNAs. By targeting its own mRNA, HIV-1 induces its own latency [108, 109]. Moreover, HIV-1 can also suppress the miRNAs-mediated silencing pathway by reducing the expression of miRNA-17, miRNA-5p, and miRNA20a that results in increased expression of Tat cofactor PCAF ultimately enhancing the viral transcription [110].

HIV-1 products interfere directly with the cellular RNAi machinery through different mechanisms. Firstly, Tat physically interacts with the helicase domain of Dicer and partially represses the ability of Dicer to process precursor dsRNA into small interfering RNAs (siRNAs) [111, 112]. Further, the viral TAR sequence prevents the formation of a functional RNA-induced silencing complex (RISC) by sequestering the Dicer-interacting protein TAR RNA-binding protein 2 (TRBP2) [113]. Finally both cellular and and viral mRNAs could be involved in maintaining HIV-1 latency or in controlling low ongoing viral replication [114].

## 3. Cellular Reservoirs in HIV-1 Pathogenesis

 HIV-1 uses different strategies to survive within infected individuals. The macrophages, dendritic cells (DCs), and CD4+ T lymphocytes are considered reservoirs for HIV-1 infection. In CD4+ T cells, the viral replication is dependent upon the cell cycle of the host cell and HIV-1 entry into activated CD4+ T lymphocytes leads to productive infection. Virions found within monocyte-derived macrophages persist and retain infectivity for weeks, thus providing an environment for viral persistence. Dendritic cells capture and internalize extracellular virions via DC-SIGN which can be subsequently transmitted to T cells *in trans*. HIV-1 hidden in DCs and macrophages certainly play an important role for viral spread and cell-to-cell transmission, and its involvement in long-term viral persistence will be discussed here.

### 3.1. Monocyte-Macrophage Lineage as Viral Reservoirs

 Cells of myeloid lineage including monocytes, macrophages, and dendritic cells are the first line of defense against pathogenesis, because these cells are critical immune cells responsible for a wide range of both innate and adaptive immune functions [28, 115]. These cells are important viral reservoirs and responsible for the dissemination of HIV-1 into sanctuaries such as brain.

 Circulating monocytes are recruited to different tissues, differentiate into macrophages, and form the HIV-1 reservoirs. Furthermore, a minor monocyte subset, the CD16+ is more permissive to infection than the more abundant CD14+ CD16− monocytes subset, which account for less than 1% circulating monocytes [116]. Macrophages contain the CD4 receptor and CCR5 and CXCR4 coreceptors which are early cellular targets for HIV-1. These cells are able to produce and harbor the virus for longer period of time due to high resistance to cytopathic effects [117]. The resident macrophages of central nervous system like microglial cells are involved in the pathogenesis of HIV-1-associated dementia, survive for many years, and are potential reservoirs for HIV-1 [118, 119].

 Macrophages can harbor large quantities of unintegrated viral DNA in circular form, which remains unstable for up to two months in nondividing macrophages [120, 121]. Further the viral protein Vpr is important for viral replication in monocyte macrophages lineage but not in nondividing CD4+ T cells. The deletion of Vpr decreases the transcription from unintegrated HIV-1 DNA up to 10 times [122, 123]. A recent finding shows that infected human macrophages can support persistent transcription from this unintegrated DNA which suggests that these circular forms of episomal DNA may therefore account for persistence and expression in nondividing cells such as macrophages [124, 125]. However, several mechanisms generating HIV-1 postintegration latency have been described in the macrophages, including lack of functional Tat, availability of host transcription activator or repressors, influence of chromatin environment, and host antiviral processes such as miRNAs [126].

 Another strategy that allows the virus to infect and persist in macrophages is the resistance to apoptosis. The NF-*κ*B pathway is activated upon HIV-1 infection in primary monocytes and macrophages [127, 128]. It has been proposed that TNF-*α*-induced NF-*κ*B activity might be involved in the inhibition of apoptosis and the survival of monocytes and macrophages. NF-*κ*B-mediated resistance to TNF-*α*-induced apoptosis might result in a decreased susceptibility to apoptosis of macrophages versus T cells in the context of chronic immune activation during HIV-1 infection [129]. Further, the absence of apoptosis in HIV-1-infected primary macrophages has been correlated with an increase in antiapoptotic Bcl-2 and Bcl-XL proteins and a decrease of proapoptotic Bax and Bad proteins [130]. Furthermore, macrophages express 10 times lower number of cell surface CD4 receptor than CD4+ T cells and therefore are less susceptible to HIV-1 superinfection [131]. High number of CD4 receptors in HIV-1-infected CD4+ T cells induce a dramatic reduction in the infectivity of release virions by sequestering the viral envelope by CD4, while the less number of CD4 on the cell surface of the macrophages might favor the release of infectious virions from infected cells and thereby could optimize the transmission of virions to cells present in the vicinity [132]. Further the viral life cycle of HIV-1 or virion production is 6 times slower in MO than in primary T cells due to a slower reverse transcription process, allowing the MO to form long lasting viral reservoirs [133, 134].

 Dendritic cells are also involved in HIV-1 propagation, through capture of viruses by receptor DC-SIGN (DC-specific ICAM3-grabbing non integrin) as well as through efficient HIV-1 transmission to T cells at the virological synapse [135]. Follicular dendritic cells in lymphoid tissues are specialized in trapping and retaining the antigens, including HIV-1 virions, on their surface in the forms of immune complexes [136, 137]. Further, mature myeloid dendritic cells located in lymph nodes can sustain the very low virus replication and therefore have potential role in HIV-1 latency [138]. The mechanism of viral persistence in these cells is not yet clearly understood [139].

 CD34+ haematopoetic cells (HPCs) also serve as a viral reservoir, since a subpopulation of CD34+ HPCs expresses CD4 and CCR5 and/or CXCR4 and these cells are susceptible to HIV-1 infection [140, 141]. Furthermore, HIV-1-infected CD34+ HPCs have been detected in some patients where these HPCs are associated with impaired growth and development [142]. Then these HPCs generate a subpopulation of monocyte which differentiates in dendritic cells, generating an infected cell lineage that may spread HIV-1 to sanctuaries [143].

### 3.2. Lymphocytes: Source of Latently Infected Cells

 The most T lymphocytes in the body are in a resting G0 state, and following activation, these resting naïve T cells, in response to antigen, undergo a burst of proliferation and differentiation in response to antigen and give rise to effector T cells. Most of these cells die during the immune response, but a subset survive and return to G0 state and become memory T cells. These lymphocytes persist as memory cells with different pattern of gene expression for the long-term survival and rapid response to the relevant antigen in the future [144, 145].

 Indeed, the activated CD4+ T cells are highly susceptible to HIV-1-infection and die quickly as a result of cytopathic effects either of the virus or of the host immune system. However, a subset of HIV-1-infected CD4+ T cells revert back to a resting state and survive for longer period of time [38]. Both naïve and memory subpopulation of resting lymphocytes provide an extremely restrictive environment for HIV-1 replication due to low CCR5 expression, low nucleotide pools and ATP level, and cytoplasmic APOBEC3G [79, 146]. Sometime, viral DNA cannot produce viable particles in this environment, but it can generate some RNA transcript and produce HIV-1 Nef (negative factor) in resting CD4+ T cells and macrophages that could increase cell activation and facilitate viral replication, either in the same cell or in the surrounding cells through production of soluble Nef from HIV-1-infected macrophages [147, 148].

 In addition to macrophages or dendritic cells, a stable form of latency also occurs in CD4+ T cells that carry integrated provirus [149]. Certain chemokines CCL19, CXCL9/CXCL10, and CCL20 activate the cofilin and actin dynamics necessary for the development of latency in resting CD4 T cells [150]. Since the HIV-1 integration requires cell activation to allow efficient reverse transcription and nuclear import of preintegration complex [151], the postintegration latency occurs when infected activated T cells return to quiescent or memory cells. The phenotypes of these resting T cells carrying a nonproductive HIV-1 infection have specific set of surface markers such as CD4+, CD25−, CD69−, and HLA-DR− [152]. Further, it has been estimated to comprise 10^6^–10^7^cells in asymptomatic patients, whose infected naïve CD4+ T cells can harbor an average of 3 to 4 copies of integrated HIV-1 per cell [153]. These cells do not allow to complete viral replication unless they are activated, and their stability and long half-lives represent major obstacle to HIV-1 eradication [154].

## 4. Targeting HIV-1 Reservoirs: A New Therapeutical Approach

 The implementation of HAART therapy has improved the survival and quality of life of HIV-1-infected individual, but it has unable to eradicate the virus from latently infected reservoirs like memory CD4+ T cells and macrophages constituting a major obstacle in HIV-1 eradication [155]. The frequency of HIV-1-infected cells, in the patients on HAART, has been reduced to less than one cell per 10^6^ resting CD4 T cells, but after many years of treatment, the frequency of these infected cells is not decreasing further [152, 156]. Moreover, some reservoirs are found in tissue sanctuary sites, like the brain, that are protected from drug penetration [157].

 Today, the current HIV-1 therapy has failed to demonstrate significant and persistent decline of these latent reservoirs, which appears small but stable and contains both wild type and drug resistant viral species. These considerations attract HIV-1 research to search for new and original anti HIV-1 treatment strategies. Furthermore, the efforts to tackle HIV-1 latency fall into two keys: first blocking the development of latency and second reactivating the viral reservoirs in chronically infected individuals to clear the virus. These challenging targets could be achieved by targeting the viral reservoirs by HAART intensification or by using transcriptional regulators.

### 4.1. HAART Intensification

 HIV-1 reservoirs are supposed to cause persistent low levels of HIV-1 RNA at a few copies/mL that are detected in HIV-1 patients on HAART. HIV-1 RNA from these reservoirs results from ongoing low level of viral replication conveying message to HIV-1 researcher that HAART is not hard enough [158, 159]. To tackle this problem, one possible solution is the HAART intensification. The objective of HAART intensification is to achieve complete suppression of residual viremia [160]. However, recent data on HAART intensification failed to decrease the residual viremia any more than normal HAART, suggesting that current regimen can halt ongoing cycles of viral replication effectively [161]. The approval of potent drugs targeting CCR5 and integrase (raltegravir) has raised the new hope for successfully decreasing the reservoir size particularly in patients with primary infection [162–164].

### 4.2. Strategies Based on Transcriptional Inhibitors to Control HIV

 Beside the combination of HIV-1 gp41, reverse transcriptase, and protease inhibitors, new drugs should be developed to target other steps of HIV-1 life cycle [165]. For example proteins involved in the transcription of proviral genome could be targeted. Further the drug could be designed to target cellular cofactors or viral protein like Tat that involves in the activation of transcription [166]. Several transcription inhibitors already characterized such as C-terminal truncated STAT5, Staf 50, prothymosin *α*, and thioredoxin reductase might be used to control the viral gene expression [167, 168]. In macrophages, the inhibition of NFAT and 5′LTR interaction by siRNA suppress the HIV-1 replication and therefore progression of AIDS also [169]. Furthermore, the treatment of HIV-1-infected lymphocyte with O-GlcNAcylation enhancing agent glucosamine represses viral transcription thus opening the way to metabolic treatment [170]. Further new approaches based on engineered transcription factors are now emerging with zinc finger protein as an attractive therapy for HIV-1 since their binding to HIV-1 LTR in a sequence-specific manner is associated with repression of LTR activation [171, 172]. For example, OKT18, a zinc finger protein, can reduce the HIV-1 replication by targeting the Tat-induced HIV-1 LTR activity. Interesting, zinc-finger protein also has the ability to influence the chromatin and nuclear organization through protein involved in epigenetic regulation [173, 174].

 The HIV-1 proteins (Tat, Nef, gp 120) should be also targeted, as these proteins have critical functions in different steps of viral life cycle and also in the acquired resistance to apoptosis. A better understanding of mechanism involved in resistance to apoptosis, has also allowed to devise new drugs against host factors which render the cells susceptible to die [175]. For example, the chemokine receptor CCR5, involved in virus entry and apoptosis could be targeted [176]. Further a chemotherapeutic drug, Imatibib, restored apoptotic sensitivity of HIV-1 macrophages through inhibition of activity of the prosurvival cytokine macrophages colony stimulating factor [177]. Finally, the addition of Akt inhibitors (Miltefosine) is also promising molecules for targeting long-lived viral reservoirs [178].

### 4.3. HIV-1 Reactivation from Latent Reservoirs

 A new strategy so-called shock and kill has been recently proposed to eradicate the virus from infected patients. The main objective is to facilitate the reactivation of viruses from the latent reservoirs, naturally (via host immune system or viral cytopathic effects), which are then destroyed by HAART [16, 179]. Many factors have been involved in reactivation including physiological stimuli, chemical compounds (phorbol esters), HDACIs (histone deacetylase inhibitors), p-TEFb activators, and activation with antibodies (anti-CD3). Several eradication protocols passed through preclinical studies but to date all failed in clinical trials [180]. The combined therapy with IL-2 and HAART has not reduced the HIV-1 reservoirs, and viral rebound has been systematically observed. A combination of antibodies (anti-CD3) and IL-2 has proved to be highly toxic and is not further advised for HIV-1 treatment [181]. In addition, IL-7 can reactivate HIV-1 from latency *in vitro* through the induction of JAK/STAT signaling pathways. IL-7 increases the TCR repertoire and induces the proliferation of both naïve and memory T cells, making this cytokine an attractive candidate for future study [182].

 The use of antibodies coupled to drugs and treatment with immunotoxins are also proposed strategies for selective killings of infected cells. The combination of immunotoxins and viral reactivation agents has cleared HIV-1 in cultures of lymphocyte from patients and also in animal model. Unfortunately the toxic side effects of this treatment precluded it for further development [183]. Furthermore, HDAC inhibitors or DNA methylation inhibitors are an attractive potential means of inducing broad reactivation of HIV-1 reservoirs. The combination of TSA (an HDACI) and TNF-*α* (NF-*κ*B inducer) synergistically activates the HIV-1 promoter. However, toxicity of these compounds undermines their clinical interest for HIV-1 therapy [184]. Although promising results in the reduction of HIV-1 reservoirs were reported using HDAC inhibitor VPA (valproic acid), more recent studies did not confirm these results [185, 186]. However, the inability of VPA (a week HDACI) to reactivate the latent reservoirs, when used alone, might have impact on the decay of HIV-1 reservoirs, when combined with other HIV-1 inducers (prostratin) [187]. Prostratin, a nontumorigenic phorbol ester, increases HIV-1 transcription through PKC activation and induction of NF-*κ*B and SP 1. Prostratin also downregulates HIV-1 receptors, which has the additional advantage of decreasing the risk of reinfection [188]. This compound has been advanced in clinical development, and recent synthesis made this drug available for clinical trials [189].

## 5. Conclusion

 HIV-1 infection is currently controlled by HAART but it has long-term toxicity and does not eradicate HIV-1 latent reservoir. It is now increasingly clear that epigenetic restriction poses an initial hurdle to viral transcription and cause of maintenance of viral latency. HIV-1 latency is regulated by both cellular and viral factors. A better understanding of epigenetic regulation of HIV-1 latency and identification of new pharmacological targets would open the doors to clear the viral reservoirs.

## Figures and Tables

**Table 1:** Licensed antiretroviral drugs.

Name	Trade name	Company	Launched
Nucleoside/nucleotide reverse transcriptase inhibitors			

Zidovudine	Retrovir	GlaxoSmithkline	1987
Didanosine	Videx	Bristol-Myers Squibb	1991
Zalcitabine	HIVID	Roche	1992
Stavudine	Zerit	Bristol-Myers Squibb	1995
Lamivudine	Epivir	GlaxoSmithkline/shire pharmaceuticals	1998
Abacavir	Ziagen	GlaxoSmithkline	1999
Tenofovir	Viread	Gilead	2001
Emtricitabine	Emtriva	Gilead	2003

Non-nucleoside reverse transcriptase inhibitors			

Nevirapine	Viramune	Boehringer Ingelheim	1996
Efavirenz	Sustiva, Stocrin	Bristol-Myers Squibb, Merck	1998
Delavirdine	Rescriptor	Pharmacia, Upjohn, Agouron, Pfizer	1999
Rilpivirine	Edurant	Tibotec Therapeutics	2011
Etravirine	Intelence	Tibotec Therapeutics	2008

Protease inhibitors			

Saquinavir	Invirase	Hoffmann-La Roche	1995
Indinavir	Crixivan	Merck	1996
Ritonavir	Norvir	Abbott, GlaxoSmithkline	1996
Nelfinavir	Viracept	Agouron, Pfizer	1997
Amprenavir	Agenerase, Prozei	Vertex	1999
Lopinavir + Ritonavir	Kaletra, Aluvia	Abbott	2000
Atazanavir	Reyataz, Zrivada	Bristol-Myers Squibb, Novartis	2003
Fosamprenavir	Lexiva, Telzir	Vertex, GlaxoSmithkline	2003
Tipranavir	Aptivus	Boehringer Ingelheim	2005
Darunavir	Prezista	Tibotec	2006

Entry inhibitors			

Enfuvirtide	Fuzeon	Trimeris, Roche	2003
Maraviroc	Celsentri, Selzentry	Pfizer	2007

Integrase strand transfer inhibitors			

Raltegravir	Isentress	Merck & Co., Inc.	2007

Multi-class combination inhibitors			

Efavirenz, Emtricitabine, and Tenofovir disoproxil fumarate	Atripla	Bristol-Myers Squibb and Gilead Sciences	2006
Emtricitabine, Rilpivirine, and Tenofovir disoproxil fumarate	Complera	Gilead Sciences	2011
